# Disambiguating Multi–Modal Scene Representations Using Perceptual Grouping Constraints

**DOI:** 10.1371/journal.pone.0010663

**Published:** 2010-06-09

**Authors:** Nicolas Pugeault, Florentin Wörgötter, Norbert Krüger

**Affiliations:** 1 Centre for Vision, Speech and Signal Processing, University of Surrey, Guildford, United Kingdom; 2 Bernstein Center for Computational Neuroscience, Göttingen, Germany; 3 Maersk McKinney Moller Institute, University of Southern Denmark, Odense, Denmark; National Microelectronics Center, Spain

## Abstract

In its early stages, the visual system suffers from a lot of ambiguity and noise that severely limits the performance of early vision algorithms. This article presents feedback mechanisms between early visual processes, such as perceptual grouping, stereopsis and depth reconstruction, that allow the system to reduce this ambiguity and improve early representation of visual information. In the first part, the article proposes a local perceptual grouping algorithm that — in addition to commonly used geometric information — makes use of a novel multi–modal measure between local edge/line features. The grouping information is then used to: 1) disambiguate stereopsis by enforcing that stereo matches preserve groups; and 2) correct the reconstruction error due to the image pixel sampling using a linear interpolation over the groups. The integration of mutual feedback between early vision processes is shown to reduce considerably ambiguity and noise without the need for global constraints.

## Introduction

Both human and machine perception involve a progressive abstraction of visual information, from the raw signal provided by the eyes or the cameras towards symbolic, object–centric representations [1]. One problem endemic to visual perception is that each abstraction step requires the taking of some decision about the information, effectively interpreting it; the large amount of noise and ambiguity in the visual signal may lead to erroneous interpretations, as discussed by, e.g., Aloimonos and Shulman [2]. There exist several approaches to solve this problem. One is to design features that describe more closely the original signal, and therefore require less abstraction. However, the resulting representation only describes the appearance of image patches as well as image noise, and lacks a semantic description of shapes — useful, e.g., for grasping, robotic control, planning. Nonetheless, a large amount of work on signal processing and invariant feature descriptors [3] lead to significant progress for tasks like navigation [4] and object recognition [5]. An alternative is to extract abstract symbolic representations directly from the image. One notable attempt by Nevatia and colleagues [6], [7], makes use of a feature hierarchy for stereo reconstruction. Another notable class of systems is the model–based vision, where a large amount of world knowledge is available and is used to disambiguate and interpret the visual signal. One problem with the latter approach is that the large amount of ambiguity and noise present in images can lead an early extraction of symbolic features to fail, failures which are difficult to correct. The dilemma between those two approaches can be expressed in terms of the bias/variance dilemma in neural networks [8]. Namely, the use of sophisticated models in vision introduces more bias in the system, whereas signal based approaches lead to more variance.

In the present work, we attempt to address the above dilemma by proposing a gradual abstraction that postpones decision taking using mutual feedback between two mid–level visual processes, namely perceptual grouping and stereopsis, to reduce ambiguity and noise. Ambiguities addressed here include incorrect stereo matches and inaccurate 3D reconstructions. Moreover, properties of the local signal such as local estimates of orientation, phase and colour will also be stabilised by perceptual grouping mechanisms. This work makes use of a sparse symbolic scene representation based on multi–modal *primitives* [9]. In this work, the term ‘multi–modal’ stresses that the descriptors cover different *visual* modalities such as motion, orientation and colour; it is not meant to indicate different *sensorial* modalities. Primitives form a local feature vector containing multi–modal visual information covering appearance as well as geometric information, in 2D and 3D. Such multi–modal descriptors offer certain advantages for the representation of visual scenes. For example, they allow for the explicit formulation of visual semantics in terms of meaningful local descriptors and higher–order relations between them, such as motion, co–planarity and similarity of appearance (see, e.g., [10]). One property of symbolic representations is that the transfer of visual information to a symbolic level increases the predictiveness of visual events [11] and at the same time decreases the memory and bandwidth required to process and transfer information. Hence, in these representations, regularities between visual events can be efficiently used for disambiguation. Primitives–based visual representations are used in a variety of applications, covering, e.g., object learning [12] and grasping [13].

The contributions in this paper are threefold: first we propose a local perceptual grouping mechanism making full use of the multi–modal and semantic information carried by the visual primitives; second, we propose a stereo matching scheme for primitives, allowing for the reconstruction of the 3D equivalent of 2D primitives; third, we investigate how perceptual grouping reduces ambiguities in the reconstructed 3D representation. In the following, these contributions will be described in more detail and put into the context of related work.

This paper's first contribution is a perceptual grouping scheme making use of the multi–modal information carried by the primitives. Perceptual grouping can be divided into two tasks: 1) defining an affinity measure between primitives and using it to build a graph of the connectedness between primitives, and 2) extracting groups, which are the connected components of this graph. We will only define the affinity measure between primitives, and not extract the groups themselves explicitly, as we only need a primitive's local grouping information to apply the correction mechanisms proposed in this paper. Similar affinity measures have been proposed [14], [15], formalising a *good continuation* constraint, and Elder and Goldberg [16] included the intensity on each side of the contour into a Bayesian formulation of grouping. We go beyond this work by proposing a multi–modal similarity measure, composed of phase, colour and optical flow measurement, and combine it with a classical good continuation criterion forming a novel multi–modal definition of the affinity between primitives.

As a second contribution, this work extends the work by Krueger and Felsberg [17] by enriching the multi–modal stereo matching using local motion [18] and, more importantly, by evaluating statistically the importance of the different visual modalities for stereo matching using ground truth range data.

As a third contribution, we make use of perceptual groups of primitives to disambiguate stereo matching and correct the 3D scene reconstruction. Grouping allows for the interpolation of visual properties such as position, local orientation, phase and colour, and thus helps to improve local feature extraction. This paper studies how perceptual grouping information can be used to disambiguate stereopsis and 3D reconstruction using primitives. If we assume that image contours (2D) are likely to be the projection of 3D contours on the image, then we can expect all 3D contours to project as 2D contours on each camera plane (except in the case of partial occlusions). Conversely, this also implies that any contour in one image has a corresponding contour in the second image. We therefore propose a non–local *external* stereo confidence measure, which estimates how well a primitive's neighbours that belong to the same group agree with that primitive's putative stereo correspondences. This allows for discarding a large number of putative stereo correspondences, hence reducing the ambiguity of the stereo matching and scene reconstruction processes. Moreover, the interpolation of the curves described by groups of primitives is used to correct these primitives' geometric and appearance modalities.

**Figure 1:**
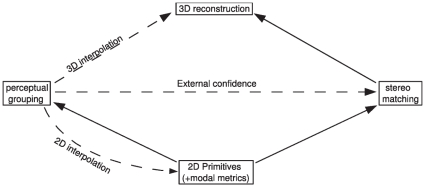
Summary of the scheme presented in this paper. In this figure, solid arrows mean direct dependencies and dashed lines corrective feedback.

The scheme presented in this paper is illustrated in Figure 1, where solid lines stand for forward dependencies and dashed lines for feedback mechanisms. The local symbolic representation is extracted from the images. From this representation, we extract perceptual groups (i.e., contours) and we use correspondences across a pair of stereo views of the scene to reconstruct a local and symbolic 3D representation of the scene, equivalent to the 2D image representations it is reconstructed from; this is the feedforward part of the scheme, represented with solid lines. Then, the perceptual grouping information is used to correct the 2D symbolic image description, the stereo matches, and the reconstructed 3D scene representation; this is the corrective part of the scheme, represented with dashed lines.

## Methods

This section is structured as follows: first, the multi–modal primitives are described; second, distance measures for all modalities are proposed; third, the grouping mechanism is presented; fourth, the stereo matching scheme is discussed; then, a scheme for increasing stereo matching reliability from grouping information is described; finally, we present a scheme to correct 2D and 3D primitives' position and orientation by interpolating the curves described by groups of primitives.

### 2D primitives

Numerous feature detectors exist in the literature (see Mikolajczyk and Schmid [3] for a review). Any feature based approach can be divided into two complementary tasks: an interest point detector [19], [20] and a descriptor encoding information from a local patch of the image at this location, that can be based on histograms [3], [21], spatial frequency [22]–[24], local derivatives [25]–[27], steerable filters [28], or invariant moments [29]. In [3], these different descriptors have been compared, showing a best performance for SIFT–like descriptors (Scale Invariant Feature Transform [21]).

The primitives we will use in this work are local, multi–modal edge descriptors, described in Ref. [9]. In contrast to the above mentioned features, primitives focus on giving a semantically and geometrically meaningful description of the local image patch. The importance of such a semantic grounding of features for a general purpose vision front–end, and the relevance of edge–like structures for this purpose are discussed by Elder [30].

In the first step, an event map and the associated local phase are computed using the *monogenic signal* [31] — note that other signal processing could alternatively be used (e.g., steerable filters [28]). The 2D primitives are sparsely extracted at locations in the image that are most likely to contain events (edges or lines); these locations are detected using the local intrinsic dimension [32]. Sparseness is assured using a classical winner–take–all operation, which guarantees that the extracted primitives describe different image patches. Multi–modal information is gathered locally from the image, including the position 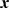 of the centre of the patch, the orientation 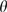 of the event, the phase 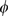 of the signal at this point, the colour 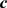 sampled over the image patch on both sides of the event, and the local optical flow 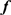 computed using the classical Nagel algorithm [33] (the flow is disregarded for still images). The phase encodes the type of contrast transition across the event, e.g., dark to bright edge or dark line on bright background. See Ref. [22]–[24]. Consequently, a primitive is described by the multi–modal vector
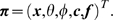
(1)


**Figure 2:**
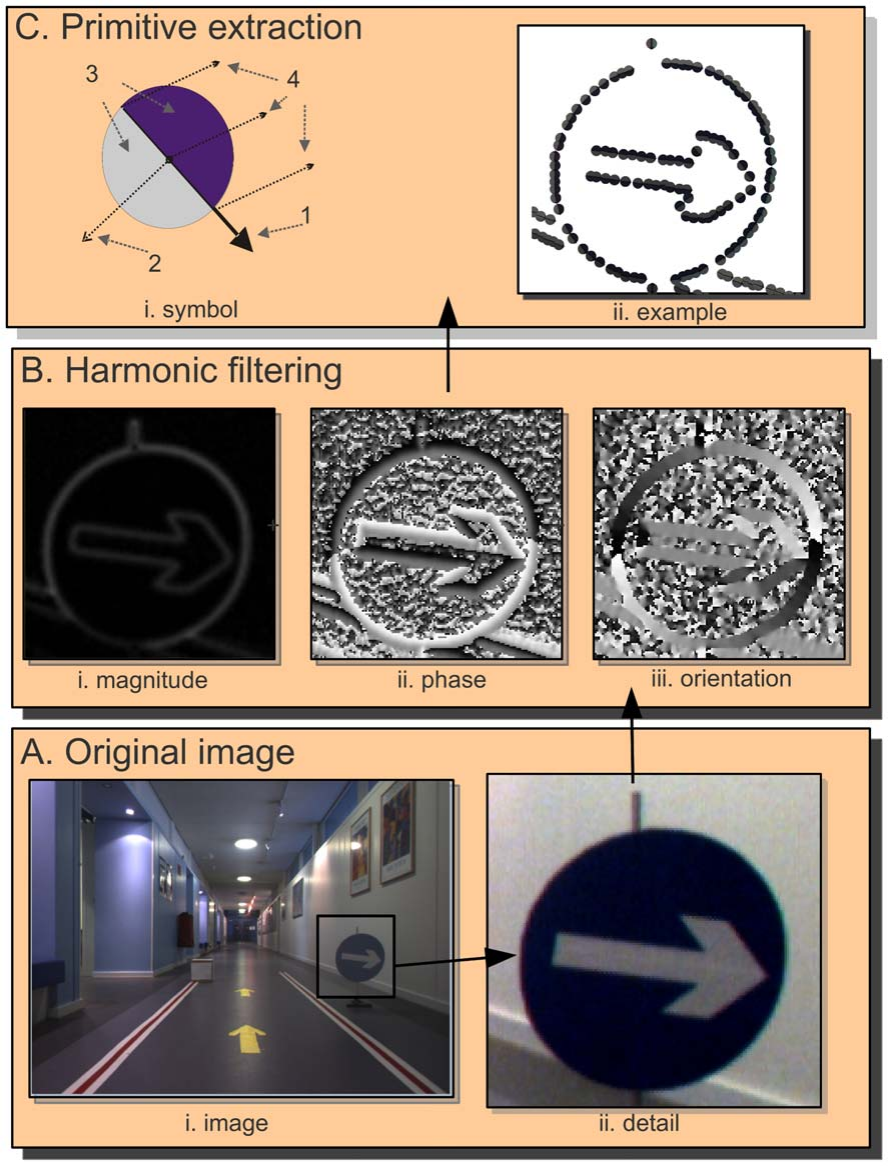
Illustration of the primitive extraction process from an indoor video sequence. **A** The original image and a magnified detail. **B** Harmonic filtering (using, e.g., Gabor wavelets, monogenic signal or steerable filters) provides estimates of the local (i) magnitude, (ii) orientation, and (iii) phase of the signal. **C** Primitive extraction: (i) the symbolic primitive, where 1 stands for the orientation, 2 for the phase, 3 for the colour, and 4 for the optic flow; (ii) example of the primitives extracted from the image detail.

The set of primitives describing an image is called *image representation* and written 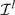 and 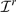 for images from the left and right camera. The image representation extracted from one image is illustrated in Figure 2. In the upper–left corner, panel A shows one image extracted from an indoor video sequence; panel B shows the result of a local filtering; and panel C shows the extracted primitives.

Note that these primitives are of lower dimensionality than, e.g., SIFT features (12 vs. 128) and can therefore suffer from a lesser distinctiveness (two unrelated primitives have a greater chance to have a similar aspect). Nonetheless, we will show in the results section that they are distinctive enough for a reliable stereo matching if the epipolar geometry of the cameras is known. The rich information carried by the 2D primitives can be used to reconstruct them in 3D, providing a more complete scene representation. Geometric meaning allows a description of proximate primitives in terms of perceptual grouping, as will be discussed in the following section.

### Metrics of 2D primitives

**Figure 3:**
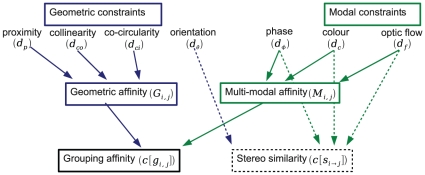
Illustration of the measures used in this paper and how they are combined. Solid arrows indicate the metrics used for stereopsis, dashed lines the metrics used for perceptual grouping.

In this section, we define metrics for each of the primitives' modalities. Those metrics will be used in the following for perceptual grouping of primitives and for stereo matching. Figure 3 illustrates how the distance measures defined here are combined. In the case of perceptual grouping (solid lines), proximity, collinearity and co–circularity measures between a pair of primitives are merged into a Geometric affinity, whereas the distances in phase, colour and optic flow form the Multi–modal affinity. The combination of those two form the overall affinity 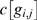 that is used to group 2D primitives. In the case of stereopsis (dashed lines) the orientation distance between the two primitives replaces the geometric criterion. Then the multi–modal similarity is computed from orientation, phase, colour and optic flow distances.

Note that, in the context of perceptual grouping, the orientation difference is replaced with a more sensible interpretation of the good continuation constraint, combining proximity, collinearity and co–circularity; in contrast, the stereo similarity makes direct use of the orientation difference.

**Orientation:** If we consider two primitives 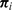 and 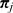, respectively with the orientations 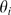 and 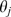, then their orientation distance is
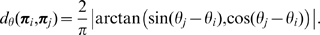
(2)The 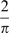 factor ensures that the orientation metric is between 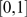, with 0 standing for parallel orientations, 0.5 for a 45 degrees angle and 1 for orthogonal orientations.

**Phase:** The phase metric 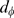 is
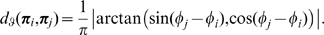
(3)The 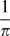 factor ensures that the phase metric is between 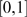, with 0 standing for two primitives encoding the contrast transition (e.g., bright to dark edge), and 1 standing for opposite contrast (e.g., a dark line and a bright line).

**Colour:** The colour metric 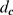 is
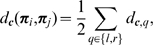
(4)where 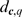 is defined in HSV space as
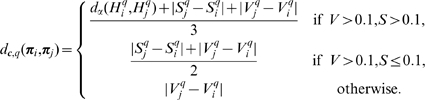
(5)Because of the conical topology of the HSV space, the hue component 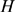 is basically random for very low saturation 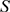, and saturation is random for low values of 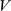. This equation discards hue information for low saturation, and saturation information for low value of 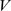, and otherwise weights evenly the colour components. In Eq. 5, 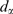 stands for the angular distance
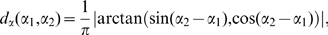
(6)and 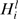 (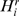), 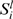 (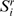) and 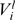 (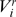) are the hue, saturation and value components on the left (right) side of the primitive 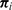.

**Optic Flow:** The optic flow 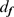 metric is
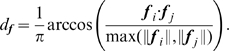
(7)


Note that these metrics are the same used in Refs. [17], [18].

### Perceptual grouping of 2D primitives

Since the 1930's, the Gestalt psychologists suggested a collection of axioms describing the way the human visual system binds together features in an image [34]–[36]. This process is generally called *perceptual grouping* and the Gestalt psychologists proposed that it is driven by properties like proximity, good continuation, similarity and symmetry, amongst others. More recently, psychophysical experiments measured the impact of different cues for perceptual grouping (see, e.g., Ref. [37]). Furthermore, Brunswik and Kamiya [38] postulated that these properties should be related to statistics of natural images. This was later confirmed by several studies [39]–[41].

We defined the primitives as local edge descriptors, and assumed that a group of primitives describes a contour in the image. The Gestalt rule of *proximity* implies that primitives that are closer to one another are most likely to lie on the same contour. According to the Gestalt rule of *good continuation*, image contours are expected to be continuous and smooth (small and constant local curvature); thus, two proximate primitives in a group are expected to be either nearly collinear, or co–circular. According to these rules, a strong inflexion in a contour will lead this contour to be described as *two* groups, joining at the inflection point. Furthermore, the position and orientation of primitives that are part of a group are the local tangents of the contour it describes. Finally, we would expect a contour's properties such as colour (on both sides) to change smoothly (or not at all) along this contour. This is formalised by the rule of *similarity*, which states that similar primitives (in terms of the colour, phase and optical flow modalities) are most likely to belong together.

The two first rules are joined into a *Geometric constraint*, that is combined with a multi–modal *Appearance constraint* into an overall affinity measure.

#### Geometric constraints

The first constraint we enforce during grouping stems directly from the symbolic quality of the primitives: primitives are local event descriptors and therefore, according to the good continuation law, they should be locally nearly collinear or co–circular to form a group. Effectively, we compute this constraint as a combination of proximity, collinearity and co–circularity measures.

**Figure 4:**
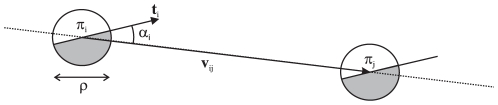
Illustration of the values used for the collinearity computation. If we consider two primitives 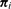 and 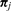, then the vector between the centres of these two primitives is written 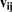, and the orientations of the two primitives are designated by the vectors 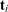 and 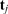, respectively. The angle formed by 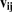 and 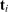 is written 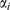, and between 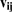 and 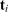 is written 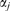. 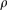 is the diameter of the primitive in pixels.

If we consider two primitives 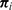 and 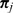 in 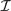, then the likelihood that they both describe the same contour 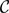 can be formulated as a combination of three basic constraints on their relative position and orientation — see Figure 4.

**Proximity:** The proximity measure is given by
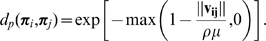
(8)Here, 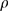 stands for the radius of the primitive in pixels, and the quantity 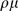 is the maximal distance between two primitives for them to be compared; more distant primitives will not be compared and therefore have a null similarity. The quantity 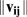 stands for the distance (in pixels) separating the two primitives' centres. We found experimentally that 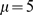 proved to be a good value — i.e., grouped primitives are distant by five timed their size at most.

**Collinearity:** The collinearity measure is
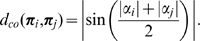
(9)


**Co–circularity:** The co–circularity measure is
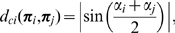
(10)where 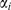 and 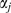 are the angles between the line joining the two primitives centres and the orientation of 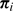 and 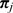, respectively (see figure 4).

**Geometric affinity:** The combination of those three criteria forms the geometric constraint:
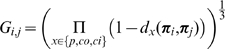
(11)where 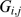 is the geometric affinity between two primitives 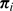 and 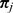. This affinity models the likelihood of a curve tangent to the lines defined by the two primitives 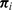 and 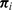; we have 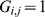 for a perfect match.

#### Appearance constraints

Effectively, the more similar the modalities between two primitives are, the more likely are those two primitives part of the same event. Note that Elder and Goldberg [39] already proposed to use the intensity as a cue for perceptual grouping, yet here we use a combination of phase, colour, and optical flow modalities of the primitives to decide, using the value of 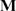, if they describe the same event.

**Appearance affinity:** The appearance–based affinity is
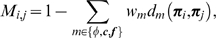
(12)where 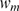 is the relative weighting of the modality 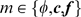, with 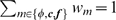, and 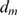 refers to the metrics defined in equations 3, 4, and 7; the modality weights were all set to 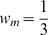; Therefore, 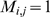 stands for a perfect match between two primitives. Because the geometric constraint models the relative orientation of two primitives in a manner more adapted to the problem of grouping line segments, the orientation metric is not part of the multi–modal constraint.

#### Overall affinity

We define this affinity from Equations (11) and (12), such that:

1. two primitives complying poorly with the good continuation rule have an affinity close to zero; and
2. two primitives complying with the good continuation rule, yet with strongly dissimilar modalities, will only have an average affinity.

**Figure 5:**
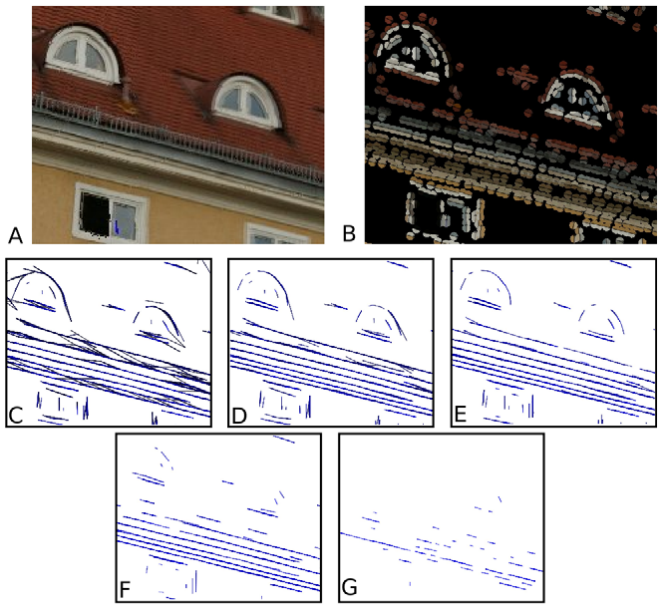
Illustration of the links extracted for different affinity thresholds. **A** detail of the original image (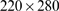 pixels); **B** extracted primitives; **C**–**G**. extracted links for values of 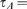 (**C**) 0.1, (**D**) 0.3, (**E**) 0.5, (**F**) 0.7, and (**G**) 0.9 — using 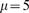. The blue lines represent the links, where more saturated lines stand for higher affinity values.

Two primitives 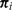 and 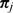 form a *link* 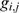 if they share a significant affinity (significant being set by a threshold on the overall affinity), and the confidence 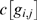 of this link is given by the overall affinity:
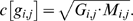
(13)We found experimentally that applying a threshold of 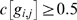 yields a good grouping, as can be seen in Figure 5.

This affinity is also a valid estimate of the likelihood for 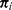 and 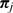 to be part of the same contour 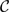. In the following, we will consider that a link 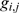 between two primitives exists if its confidence 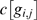 is large enough. We will call *neighbourhood* 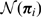 of a primitive 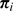 all primitives 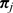 such that 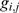 is a link:
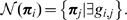
(14)


**Figure 6:**
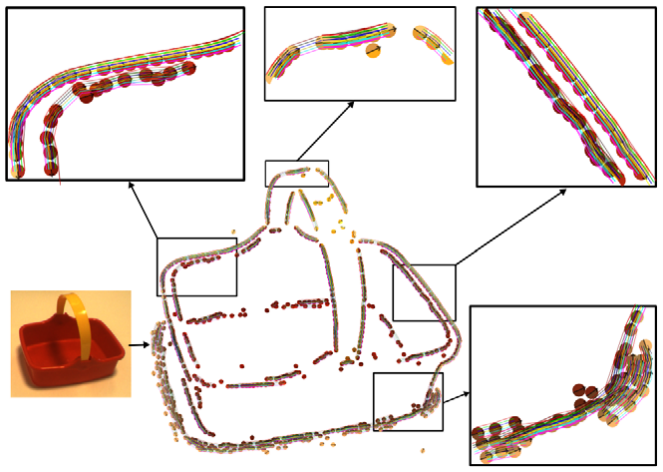
Illustration of the affinities between 2D primitives. In this figure, the 2D primitives are linked by coloured lines, where a brighter colour stands for a stronger affinity. Red stands for collinearity, green for phase, blue for colour and yellow for optical flow affinity.

Figure 6 shows the links extracted, along with the different modal affinities. The links extracted for different thresholds 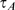 on the affinity are shown in Figure 5. In the following, links are extracted only if 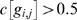. The lines in these figures describe strings of grouped primitives. One can see in these images that the major image contours are adequately described. This criterion is what is meant in the rest of the paper every time we refer to ‘groups’.

### Stereopsis using 2D primitives

In this section, we extend the concept of multi–modal primitives to 3D: first, we define a local multi–modal matching function; then we define the 3D primitives.

Classical stereopsis [42], [43] allows for the reconstruction of 3D points from pairs of corresponding points in two stereo images. A review of stereo algorithms was presented by Brown et al. [44]. Dense two–frames stereo algorithms (i.e., matching each and every pixel in the first image with a pixel in the second) were also compared by Scharstein and Szeliski [45]. The present work differs from classical approaches insofar that symbolic multi–modal entities are matched, and reconstructed, rather than points. Although it is commonplace to use complex features (e.g., SIFT) for matching, only the locations in space are generally reconstructed, whereas the present work reconstructs a symbolic local interpretation in space. The proposed method is local and makes use of the epipolar constraint to limit the scope of the correspondence search.

If we consider a 2D primitive 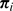 in the left image 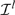, all 2D primitives 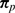 in the right image that lie nearby its epipolar line 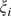 are considered as *putative correspondences*, written 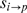. The difference between the image coordinates of 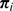 and 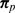 is generally called the *disparity*. We will differentiate between the orthogonal distance from the centre of 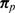 to the epipolar line 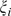, called *normal disparity*, and the distance along this line, called *tangential disparity*. The normal disparity expresses how strictly the epipolar constraint is satisfied. A certain tolerance is required here due to the representation's sparseness. In the following all primitives with a normal disparity lower than 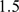 times the primitives' size are considered. The tangential disparity has a direct relation with the depth of the reconstructed 3D primitive: a tangential disparity of zero means that the point is infinitely far, whereas larger disparities denote closer points.

Finally, one putative correspondence 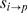 is chosen using a local winner–take–all scheme: all putative correspondences 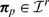 (in the right image) of a primitive 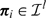 (in the left image) are competing against each other. The confidence in each of them is set to their *similarity* with the left primitive 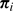, and the most similar correspondence is selected. This similarity measure is explained in the following section.

#### Multi–modal stereo similarity

The multi–modal distance between two primitives is defined as a linear combination of the modal distances between two primitives. This similarity is akin to the multi–modal affinity defined in Equation (12) with the addition of the orientation similarity, that is used here to replace the geometric constraint:
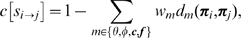
(15)where 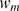 is the relative weighting of the modality 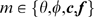, with 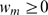 and 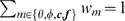. The performance of a winner–take–all stereo matching scheme based on this multi–modal similarity is evaluated on several stereo sequences in the results section.

#### Reconstruction of 3D primitives

**Figure 7:**
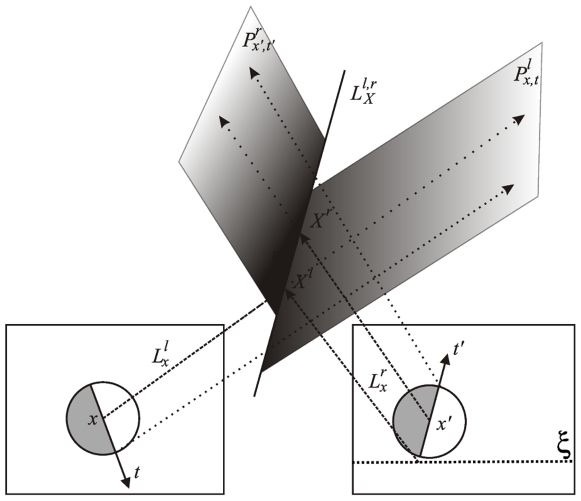
Illustration of a 3D primitive reconstruction from a stereo pair of 2D primitives. Each 2D primitive defines an image line, that back–projects as a plane in 3D space. The intersection of these two 3D planes yield a line in space that defines the 3D primitive's orientation. The 3D primitive's position is given by the intersection between the back–projections of both 2D primitives' position. We refer to [46] for a complete discussion of the 3D primitives reconstruction.

We propose to reconstruct the 3D equivalent of a stereo pair of corresponding 2D primitives, hereafter called *3D primitives* (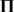) as encoded in the vector:
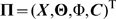
(16)where 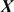 is the location in space, 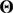 is the 3D orientation of the edge, 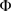 is the phase across this edge, and 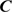 holds the local colour information on both sides of the contour. Figure 7 illustrates the reconstruction of a 3D primitive from a stereo pair of corresponding 2D primitives. A 2D primitive defines an image line, that back–projects as a 3D plane; the intersection between the two planes back–projected by the corresponding primitives provide a 3D line, onto which the 3D primitive lies. This line's orientation give the 3D primitive's orientation; its position is given by the intersection between the line back–projected by the first 2D primitive's position, and the plane back–projected by the corresponding 3D primitive. We refer to [46] for a complete discussion of the 3D primitives reconstruction.

The reconstruction shown corresponds to a multi–modal winner–take–all matching (using equation (15)) with a similarity threshold set to 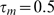.

#### Perceptual grouping of 3D primitives

In order to allow for reasoning in the 3D space, we extend the perceptual grouping defined for 2D primitives to the reconstructed 3D primitives.

Two 3D primitives 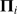 and 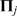 are linked 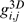, if and only if their projection in both image planes (respectively 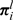 and 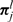 on the left image and 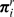 and 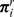 on the right) are linked (such that the two links 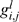 and 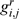 both exist), according to the logical implication
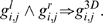
(17)This definition extends naturally the perceptual groups defined in the image domain to the 3D space.

### Perceptual grouping constraints to improve stereopsis

In this section, we define a semi-global stereo matching function that is based on the expected consistency between grouping processes in the left and right image as well as the stereo matching process. We show that matching can be improved significantly by using such kind of context information. It also allows for the establishment of groups in 3D for which additional interpolation processes can be applied to further improve the precision of reconstruction.

Because the primitive–based image representation used in this work samples lines and step–edges, it carries redundant information along contours. This redundancy can be used for constraining the stereo matching problem, leading to the two following constraints:

**(C1) Isolated primitives are likely to be unreliable:** As primitives are extracted redundantly along the contours, conversely an isolated primitive is likely to be an artefact and hence isolated primitives can be neglected.
**(C2) Stereo consistency over groups:** If a set of primitives forms a contour in the first image, the *correct correspondences* of these primitives in the second image also form a contour (notwithstanding pathological cases).

**Figure 8:**
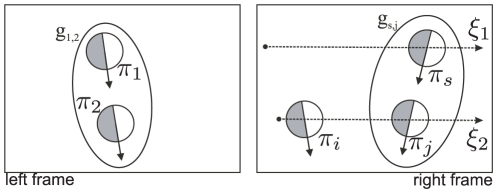
The BSCE criterion. Let 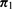 be a primitive in the left frame forming a group with a second primitive 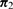. 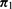 has a stereo correspondence 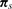 that lie on the epipolar line 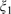 in the right image. Both 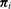 and 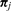 in the right image lie on the epipolar line 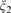 of 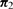; hence these two primitives are both putative correspondences of 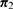.

In our representation, contour information is encoded by the link network that is the result of the perceptual grouping mechanism presented earlier; this is illustrated in Figure 8. In this figure, the orientation of the primitive 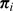 makes it the most similar (according to Equation (15)) to 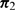; hence, the stereo correspondence 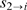 holds a higher confidence than, e.g., 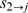. However, the putative correspondence 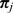 forms a group 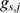, thus preserving the group relation 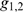 across stereo, whereas 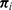 is not grouped with 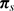. Therefore, 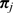 is more likely to be the true stereo correspondence of 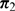.

#### Basic Stereo Consistency Event (BSCE)

Primitives represent local estimators of image contours; a constellation of primitives describes a contour as a whole. Such contours are consistent over stereo, with the notable exception of occlusion cases. As we have defined the likelihood for two primitives to describe the same contour as the affinity between these two primitives, we can rewrite the previous statement as:

**Definition 1** *Given two primitives* 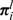 *and* 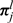 *in the left image* 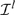 *and their respective correspondences* 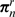 *and* 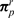 *in the right image* 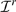 *; if* 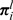 *and* 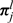 *belong to the same group in* 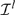 *, then* 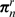 *and* 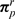 *should also be part of a group in* 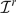 *.*

The link conservation between a pair of primitives and the stereo correspondences thereof is called *Basic Stereo Consistency Event* (BSCE) [47]. This condition can then be used to test the validity of a stereo hypothesis. Consider a primitive 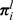, a stereo hypothesis
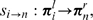
(18)and a 2D primitive 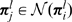 in the neighbourhood of 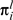 (as defined in Equation (14)), such that the two primitives share an affinity 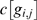 — see Equation (13). For this second primitive, a stereo correspondence 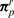 with a confidence of 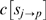 exists. We can now define an estimate of how well the stereo hypothesis 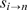 reflects the BSCE by:
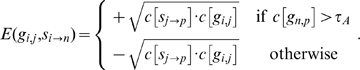
(19)


In other words: the BSCE between a primitive in the first image and one of its neighbours is high if they share a strong affinity and if both primitives' stereo correspondences in the second image *also* share a strong affinity; it is low if they share a strong affinity yet their stereo correspondences in the second image do not. This naturally extends the concept of group into the stereo domain.

#### Neighbourhood consistency confidence

Equation (19) tells us how a primitive's stereo correspondence is consistent with our knowledge of one of its neighbours' stereo correspondence. In this section we extend this definition to the whole primitive's neighbourhood. If we consider a primitive 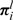 and an associated stereo correspondence 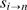, we can integrate this BSCE confidence over the neighbourhood of the primitive 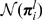 — as defined by Equation (14) —
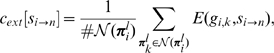
(20)where 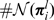 is the size of the neighbourhood — i.e., the number of neighbours of 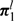 considered. We call this new confidence the *external confidence* in 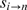, as opposed to the internal confidence given by the multi–modal similarity between the primitives — Equation (15).

### Correcting primitives using contextual knowledge

Although primitives are extracted with sub–pixel localisation, their actual accuracies vary to a large extent depending on local amounts of noise, blur and texture in the image. The primitives' position and orientation inaccuracy is amplified by stereo reconstruction [48] and can lead to large errors thereafter. Moreover, one fundamental drawback of stereo–based reconstruction of 3D shapes is that the reconstructed entities' precision decreases quickly with distance to the cameras, due to the images' finite pixel sampling [49], [50]. The symbolic quality of primitives, and groups of primitives, provides us with additional knowledge that can be used to reduce this uncertainty. Namely, groups of 3D primitives are reconstructed from pairs of 2D primitives that form a perceptual group in both stereo images, and as such, according to the grouping assumption, they describe a smooth and continuous contour of the scene (except in some pathological perspectives). This knowledge that the group as a whole should form a smooth contour can be used to correct the individual 3D primitives modalities. In this section, we propose a scheme for correcting 2D– and 3D primitives by locally interpolating the contours described by groups of primitives.

#### Triplets of primitives

If we consider three primitives 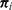, 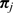 and 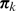, which belong to the same group, and if 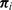 lies in between 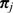 and 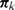 — such that the Euclidean distances between 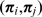 and 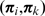 are both smaller than that between 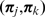 — then we call 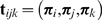 a *triplet*. Formally,
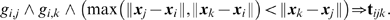
(21)


Triplets of 3D primitives can be defined in the exact same manner in 3D space: as for the 2D case, a 3D triplet 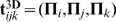 is constituted of a central primitive 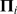 linked to two supporting primitives 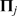 and 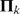, such that the central primitive lies in between the two supporting primitives (i.e., the Euclidean distances between 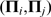 and 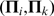 are both smaller than 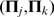). Formally,
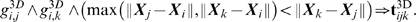
(22)These triplets are useful because it is possible to interpolate the curve between two primitives, and therefore, we can use the curve interpolated between the two supporting primitives of the triplet (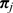 and 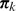) to correct the central primitive (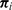).

#### Interpolation of modalities

We interpolate the curve between two (2D or 3D) primitives using Hermite polynomials [51]. These are convenient in this context as they allow for the interpolation of a curve from only two data points and the curve tangents at those points. Also, Hermite splines can be applied to interpolate 2D or 3D curves indifferently.

**Position and orientation:** The curve interpolated between two primitives 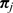 and 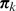, with positions 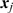 and 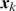, and local tangents (defined by the primitives' orientations) of 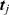 and 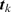 is defined as all the points 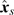 in the image, with 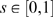 such that 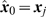 and 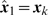 and
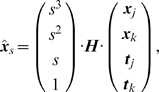
(23)where 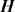 is the matrix formulation for the Hermite polynomials
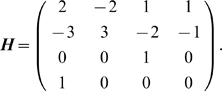
(24)


Analogously for the orientation we have
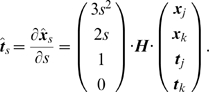
(25)


Note that the exact same formulae are used for interpolating curves between 3D primitives, but applied to 3 dimensions instead of 2.

The other modalities are interpolated by assuming that these change linearly with 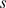 between 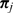 and 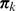:

**Phase:** The phase modality of the primitive interpolated for 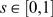 is computed as by
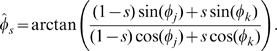
(26)


**Colour:** The colour of the interpolated primitive is computed using the following equation:
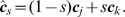
(27)


#### 2D Primitive correction

We can then correct the *extracted* primitive 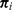 between 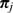 and 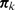 with the *interpolated* primitive 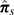. This is done for each modality 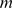 using a weighted mean between the two values. For position and colour information 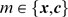, the corrected value 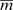 is computed by
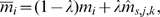
(28)where 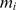 is the extracted modality value, 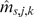 is the value interpolated at 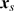 between 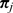 and 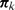, and 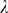 is the correction rate.

For orientation and phase 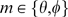, we have:
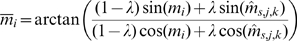
(29)


Note that in the case of 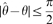, we need to operate a switch of the primitive's interpretation of the orientation as defined in Ref. [9] before correcting the orientation, colour and phase.

The correction (in Equations 28 and 29) is applied for 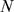 iterations, with a correction factor 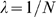. This is evaluated on an artificial scene with precise 3D ground truth in the results section, and the results showed that a small number of iterations can already considerably improve accuracy.

#### 3D primitive correction

In the 3D case, the primitives also suffer from the uncertainty that originates from the stereo matching and reconstruction processes. The 3D primitives' position in space is corrected to
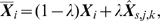
(30)and the orientation to
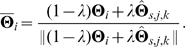
(31)This correction is applied iteratively 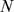 times, with a correction factor 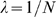. Also in this case, the results section shows that a small number of iteration suffice to improve accuracy.

## Results

This section contains an evaluation of the different mechanisms presented above. In order to evaluate the performance of the different algorithms, we used stereo video sequences generated from a high resolution images of a urban scenes, with the associated depth ground truth provided with range scanner.

**Figure 9:**
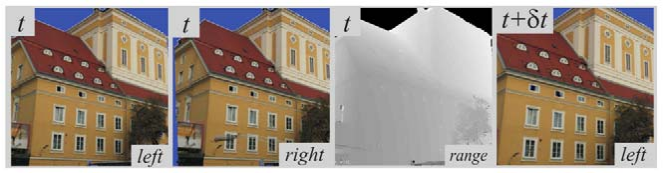
Illustration of how a sequence is generated from colour range images. The images show the first 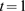 left and right images, the left disparity image, and the last left image (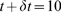).

The range scanner provided us with a single high–resolution image with associated range information, and therefore each pixel of the image is given by
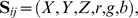
(32)where 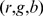 is the pixel's colour and 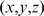 is the corresponding 3D point (according to the range scanner). For each image, we then define ten virtual pairs of stereo cameras with resolution 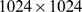, and used projective geometry to transform the original image pixels into the virtual cameras' images, then the colour of each pixel in the virtual images is linearly interpolated from the nearest 4 transformed points. The disparity between the two virtual stereo views is also linearly interpolated at all pixel positions — see Figure 9.

**Figure 10:**
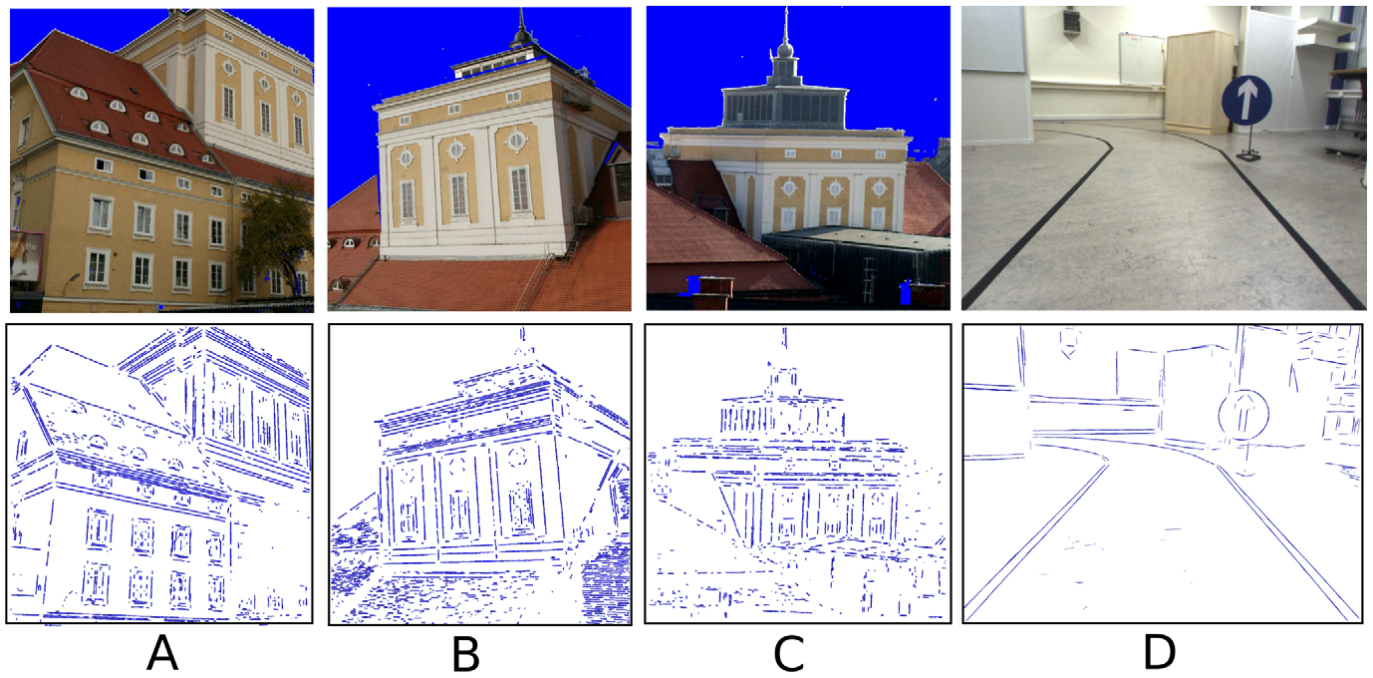
The four sequences on which we tested our approach. The top row shows one image from each sequence, and the bottom row shows the groups created.

This offers realistic video sequences with an accurate 3D ground truth. Some images generated from three different range images are illustrated in Figure 10A, B and C; the dark blue areas (like the sky) correspond to where there was no range data available, and therefore the colour cannot be interpolated. No range data was available for sequence D, therefore we only have a qualitative evaluation on this sequence.

### Stereo Evaluation

We first assessed the performance of the stereo matching scheme using each modal distance individually, plus the proposed multi–modal distance. We used the sequences with ground truth in Figure 10A, B, C to evaluate quantitatively the efficiency of each measure for stereo matching. We considered that a match was correct if its disparity error with the ground truth was smaller than the 2D primitives' size — this ensures that no erroneous match is considered as correct.

**Figure 11:**
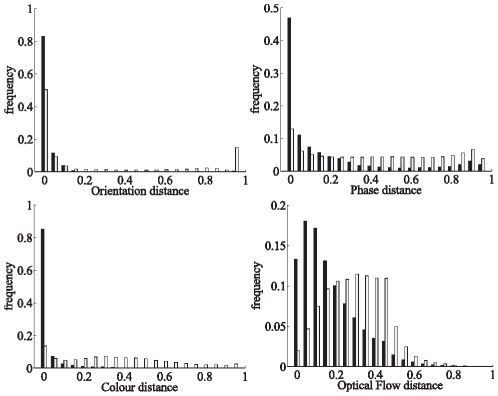
Histograms of the modal distances. Each plot shows the histograms of one modal distance (0 for identity and 1 for dissimilar items), for correct (black bars) and false (white bars) correspondences. The modal distances between putative stereo pairs are binned along the horizontal axis, and the vertical axis shows the frequency of occurrence of this value, between 0 and 1 (such that the cumulated heights of black and white bars are both 1). The histograms are computed across all three sequences in Figure 10 **A**, **B** and **C**.

**Figure 12:**
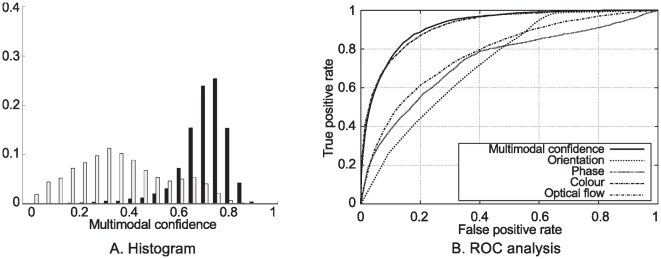
Evaluation of the multi–modal stereo. **A** Histogram of the multi–modal similarities between correct (black bars) and false (white bars) potential correspondences. **B** ROC curves for the different modalities. These results have been collected over 10 frames of the sequences Figure 10 **A**, **B** and **C**.

Figure 11 shows the histogram distributions of the modal distances between primitive pairs satisfying the epipolar constraint — for all images in sequences A, B and C. All histograms show a separation between the distributions of correct (black) and false (white) correspondences. In the phase (Figure 11 top–right) and colour (Figure 11 bottom–left) histograms, the correct correspondences show a sharp peak at a modal distance of zero, whereas the false ones display an even distribution along all distances between 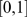. In the orientation histogram (Figure 11 top–left), the large peak at zero distance for false correspondences is explainable by the presence of parallel structures in the image. Consequently, if one draws a horizontal line in the image, this line would cross parallel contours of very similar local orientation. The optical flow distribution shown in Figure 11 bottom–right has a peaked distribution centred at a distance of 0.1 for the correct correspondences, with a long tail until 0.6. The fact that the distribution peaks at 0.1 is explained by the projective difference in the optical flow between the two stereo images (the flow is likely to be similar, but not equal); this long tail is likely to be a consequence of the noisiness of optical flow data. The false correspondences also show a broad distribution around a modal distance of 0.3; the fact that the distribution is not centred at 0.5 is a consequence of statistical distributions of edges in natural images: horizontal and vertical edges are more likely, and therefore horizontal and vertical flow vectors are also more likely. In spite of this large overlap, optical flow distance is still better than chance for identifying correct stereo correspondences from erroneous ones — see ROC analysis in Fig. 12B: the optic flow curve is above the diagonal line that indicates chance performance in ROC curves. Figure 12A shows the multi–modal similarity histogram for correct and erroneous stereo matches. There is little overlap between the two distributions, showing that the multi–modal similarity is a good criterion for stereo matching.

In order to evaluate the performance of each distance measure for the task of identifying correct stereo matches from erroneous ones, we drew the Receiver Operating Characteristic (ROC) curves for each of them. If we consider a set of putative stereo correspondences, provided that we have a distance measure for all of them and that we know from the disparity ground truth which ones are correct, it is possible to compute the ratios of correct and erroneous pairs of primitives with a distance below threshold, respectively called *true* and *false positive rates*. A ROC curve records the true positive rates against the false positive rates obtained when considering one distance measure for a sample of threshold values ranging from 0 to 1. Therefore, a random measurement would generate a nearly diagonal ROC curve, whereas a measurement that is very significant for the task would have a large area below its ROC curve. In Figure 12B, such ROC curves show the performance of the stereo matching. Each of the curves shows the performance when using each modal similarity, or the multi–modal similarity proposed in Equation (15). In this figure, we can see that the colour modality is a particularly strong discriminant for stereopsis. This is explained by the fact that the hue and saturation are sampled on each side of the edge, leading to a 4–dimensional modality (if we neglect the 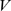 component and only keep the 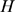 and 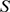), whereas phase and orientation are only 1–dimensional and optical flow is 2–dimensional (albeit the aperture problem reduces it to one effective dimension: the normal flow). Moreover, those stereo pairs of images were interpolated from a single high–resolution image with range ground truth; thus, pixel colour is consistency is unaffected by illumination and therefore artificially high between left and right images. On the other hand the poor performance of the optic flow modality could be explained by the relative simplicity of the motion in this scene: a pure forward translation of the camera, with no moving objects. Therefore, we would expect the performance of individual modalities to vary depending on the scenario, and the robustness of the multi–modal constraint could be further enhanced by a contextual weighting. Nevertheless, in a variety of scenarios the use of a static weighting proved robust enough to obtain reliable stereopsis. These results show that (1) the similarity measures in all modalities are efficient (i.e., better than chance) indicators for stereo matching, (2) the multi–modal similarity yields a better classification.

### External Confidence Threshold

In a second set of experiments, we evaluated the effect of setting a minimal threshold on the external confidence. The external confidence threshold was always applied in conjunction with a sensible threshold on the multi–modal similarity of 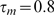.

**Figure 13:**
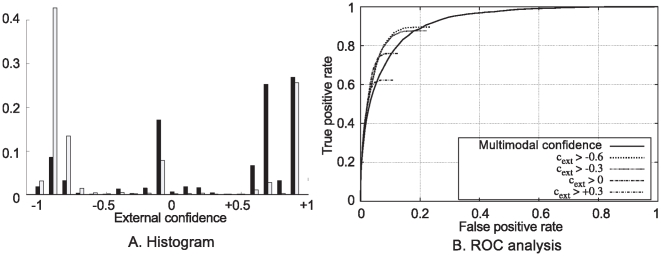
Evaluation of the external confidence. **A** Histogram of the external confidence rating for correct (black bars) and false (white bars) correspondences. **B** Each curve stands for a the application of a different threshold over the external confidence, prior to the ROC analysis. These curves represent the statistics over 10 frames of the three sequences with ground truth — see Figure 10 **A**, **B** and **C**.

In Figure 13A, one can see that the correct (black) correspondences have mostly positive external confidences, while incorrect (white) ones have mainly negative values (large peak at 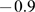). The small peak of correct correspondences for negative external confidence (near 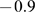) is due to the few cases where most primitives on a contour have an erroneous correspondence, and therefore the few correct ones are strongly contradicted. The large values of erroneous correspondences with external confidences of 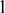 comes from repetitive structures in the image, that require more global considerations for disambiguation. Applying a threshold on the external confidence will remove stereo hypotheses that are inconsistent with their neighbourhood, and thus reduce the ambiguity of the stereo matching. Note that selecting a threshold of zero implies the removal of all the isolated primitives (see constraint **C1**) as an isolated primitive has an external confidence of zero by definition.

Figure 13B shows ROC curves of the performance for varying thresholds on the multi–modal similarity. Each curve shows the performance for a different threshold (with threshold of 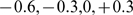, and without threshold) applied to the external confidence prior to the ROC analysis. We can see from these results that applying a bias on the decision based on the external confidence is improving significantly the accuracy of the decision process. Depending on the type of selection process desired — very selective and reliable, or more lax, but yielding a denser set of correspondences — different thresholds can be chosen. The best overall improvement seems to be reached for a threshold of 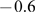 over the external confidence (with a negligible difference in performance between 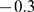 and 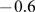). However, in the general case where a high reliability is required of the stereo matches, a small positive threshold of 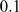 is preferred (meaning discarding all primitives which are not part of a group) is preferred. Note that when a threshold is applied to the external confidence prior to the ROC analysis, the resulting curve does not reach the 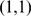 point of the graph. This is normal as the threshold already removes some stereo hypotheses even before the multi–modal confidence is considered.

Table 1 summarises the performance of the stereo matching scheme, with and without external confidence threshold (because the external confidence is within 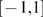, a threshold of 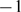 is the same as no threshold at all), on all three sequences with ground truth, showing a consistent improvement in all scenes, although the actual magnitude of the improvement varies. Sequence A, for example, contains a lot of repetitive, parallel structures which the external confidence cannot help disambiguating.

**Table 1:** Performance of the stereopsis with and without external confidence threshold.

sequence	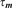	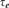	correct 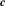	false 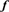	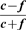
A	0.8	−1.0	3633	498	0.76
A	0.8	−0.1	3582	456	0.77
B	0.8	−1.0	2205	1178	0.30
B	0.8	−0.1	1915	447	0.62
C	0.8	−1.0	906	276	0.53
C	0.8	−0.1	804	167	0.66

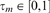
 is the multi–modal similarity threshold for stereo matching; 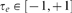 is the external confidence threshold; 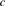 and 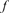 are the total number of true and false correspondences (respectively) selected by these thresholds.

**Figure 14:**
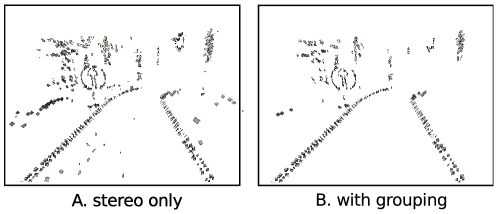
Qualitative example of the effect of the external confidence threshold. **A** primitives reconstructed from the sequence in Figure 10D, without threshold on external confidence (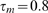, 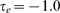). **B** primitives reconstructed from the same sequence with a threshold on external confidence (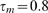, 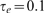).

Figure 14 illustrates the effect qualitatively for the video sequence from Figure 10D. Figure 14a) shows the 3D primitives reconstructed with a threshold on external confidence of 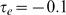. When comparing Figures 14A and 14B we can see that a large number of outliers has been discarded from the reconstructed 3D primitives, leading to a cleaner description of the scene.

### Interpolation

**Figure 15:**
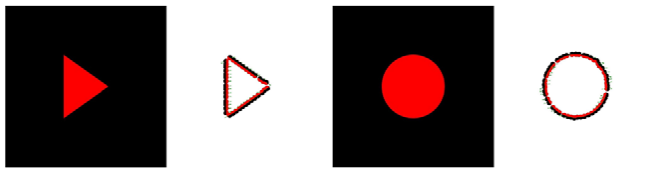
Illustration of the primitives extracted from two simple artificial sequences, featuring a triangle (left) and a circle (right). In both scenarios, the object (triangle or circle) is facing the cameras, at a depth of 100 units, the object has a radius of 10 units, and the baseline between the two cameras is 10 units. Both images shown here are from the last camera.

We evaluated the performance of the interpolation scheme, on two simple artificial sequences illustrated in Figure 15. In the case of 3D–interpolation we also evaluated the interpolation effect on the reconstructed 3D representation qualitatively. The interpolation scheme was applied for 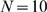 iterations, with a correction factor of 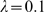.

#### 2D interpolation Results

**Figure 16:**
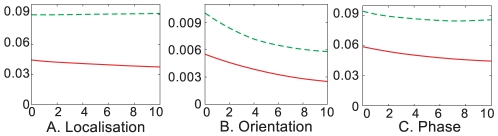
Correction of the 2D primitives using interpolation. Accuracy of the 2D primitives' localisation (**A**), orientation (**B**) and phase (**C**) after several iterations of the correction process, for the triangle (full line) and circle (dashed line) scenarios. The horizontal axis shows the number of iterations of the correction process and the vertical axis shows the error for **A** in pixels, and for **B** and **C** in radians.

The results for localisation, orientation and phase over 10 iterations of the correction process are shown in Figure 16, for the triangle (full line) and the circle (dashed line) scenarios. The horizontal axis shows the number of iterations of the correction process and the vertical axis the mean error of the 2D primitives. Note that the error is measured in pixels for the localisation and in radians for the orientation and the phase.

This sub–pixel accuracy is naturally lower for the circle scene, which is due to the contour's curvature. As primitives are local line descriptors, they can describe curved contours but they assume low local curvature. Hence, as the sub–pixel accuracy is assuming this linear model, it is performing better with purely linear structures. Nonetheless, note that the accuracy is extremely high in both cases: less than one tenth of a pixel for the localisation and and less than one hundredth of a radian for the orientation — i.e., less than 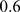 degrees.

Moreover, we note that interpolation leads to mixed results depending on the modality: we see a distinct improvement of the localisation for the triangle scene, but not for the circle scene. This is likely to be due to the use of Hermite interpolation, in two respects: first, Hermite interpolation makes use of the tangents' orientation in addition to their position; hence, the interpolated curve is sensitive to errors in orientation. Second, even if the Hermite polynomials are an efficient model for describing general curves, they do not allow a perfect interpolation of an arc; thus, interpolation at high curvature locations lead to a loss in precision. Nonetheless, the accuracy of the interpolated primitive itself is always better than the original (reconstructed by stereo).

**Figure 17:**
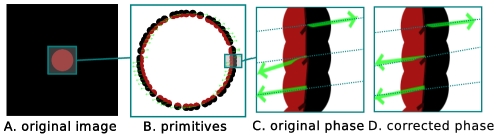
Illustration of the effect of phase correction in 2D. **A** the original image; **B** the extracted primitives; **C** detail of the primitives, the green arrows show the extracted phase, the dotted lines show the mean phase over the whole circle; **D** detail of the primitives after correction: the central primitive's phase is now closer to the dotted line.

Concerning orientation, we see a clear improvement of 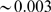 radians for both objects (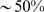 and 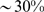 for the triangle and circle). Phase shows a clear (although smaller) improvement in both cases; the triangle scenario sees an improvement of 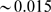 (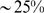), whereas the circle scenario sees an improvement of 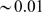 (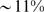). The effect of phase correction is illustrated in Figure 17. This figure shows a detail of the primitives extracted on the circle scene; the phase is illustrated on the primitives by the green arrow, which orientation indicates the phase. In this case, horizontal indicates a full contrast edge structure, and vertical a full contrast line. Figure 17C and D show the phase before and after correction, where the dotted lines show the mean phase across the whole circle. Before correction, the phase of the central primitive differs significantly from the correct one, and it is closer to the dotted line after correction.

#### 3D primitives interpolation

**Figure 18:**
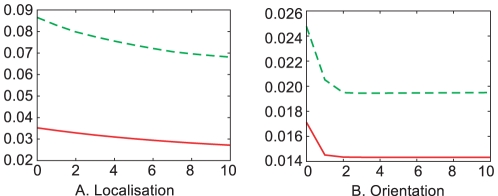
Correction of 3D primitives. Error of the **A** localisation and **B** orientation of the reconstructed 3D primitives after several iterations of the correction process. Solid lines shows the errors for the triangle scenario and dashed line for the circle scenario. The horizontal axis shows the number of iterations of the correction process and the vertical axis shows the error in **A** units (in the 3D space, arbitrary in an artificial scenario) and **B** radians.

**Figure 19:**
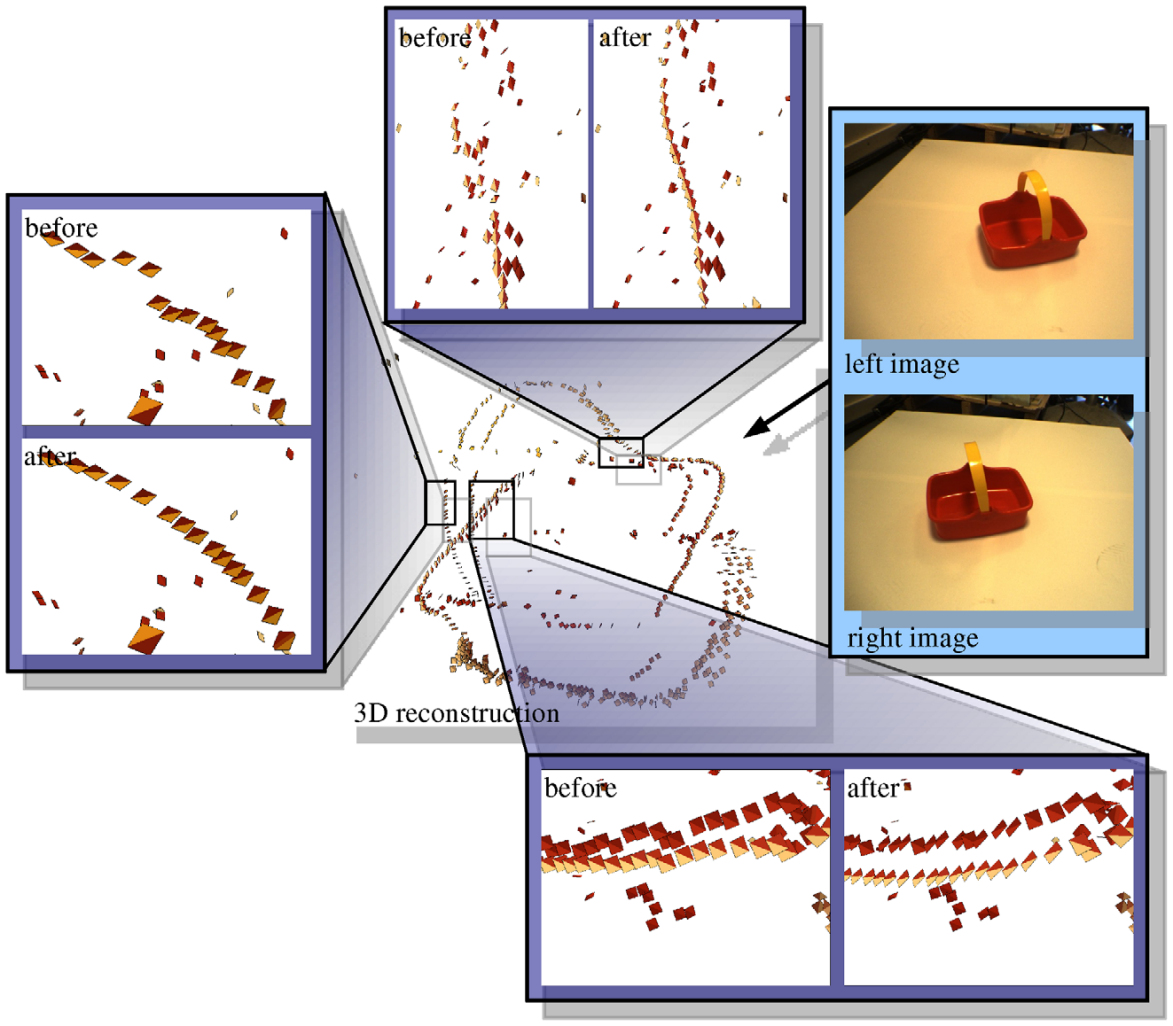
Illustration of the effect of the correction of 3D primitives using interpolation. The figure shows the reconstructed primitives before and after 10 rounds of correction, for details of an object.

This scheme was evaluated on the same triangle sequence as above (shown in Figure 15) and resulted in a reduction of the localisation error by 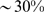; the orientation error was reduced by 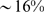 (see Table 2). When applying the same scheme to the circle scenario, the localisation error was reduced by 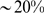; orientation error was reduced also by 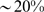 (see Table 3 and Figure 18). Figure 19 shows the effect of this smoothing on selected details in an indoor scene.

**Table 2:** Effect of the correction process on the localisation and orientation in space of the primitives reconstructed from the triangle scenario.

	localisation error		orientation error	
	mean	variance	mean	variance
before	0.03524	0.00392	0.01712	0.00082
after 10 iterations	0.02426	0.00221	0.01434	0.00056

**Table 3:** Effect of the correction process on the localisation and orientation in space of the primitives reconstructed from the circle scenario.

	localisation error		orientation error	
	mean	variance	mean	variance
before	0.08653	0.01188	0.02476	0.00071
after 10 iterations	0.06868	0.00882	0.01955	0.00046

## Discussion

In this paper, we presented several local operations on the visual primitives presented in Ref. [9], which produce a robust representation of visual scenes, some of them making use of the (still locally constrained) context.

First, we presented a simple algorithm to group primitives into contours. Contours were defined implicitly in terms of the pairwise relations between proximate 2D primitives. Note that an explicit description of the groups could easily be extracted from such an implicit definition using a variety of techniques, including: normalised [52] or average cuts [53], affinity normalisation [15], dynamic programming [54], probabilistic chaining [55], etc.

Second, we proposed to use the multi–modal similarity between 2D primitives to perform stereo matching between pairs of images. The stereo algorithm we used is purely local and therefore does not make use of global constraints (e.g., ordering constraint [56], figural continuity [57], etc.), or optimisation (e.g., dynamic programming [58], graph operations like maximal clique [59], etc.). Such global optimisations generally allow to improve significantly the performance of local stereo matching schemes, and therefore could be applied to this system to further improve the quality of stereo matching.

Third, we proposed a scheme integrating contextual information combining perceptual grouping and stereopsis to improve the reliability of the latter. The external confidence defined here is comparable to averaging over a local neighbourhood of a disparity gradient constraint along contours [60]. Also, in a similar way, Ohta and Kanade [56] proposed to apply inter–scanline consistency rules in addition to a more classical intra–scanline ordering constraint. Departing from those pixel–based constraints, the definition of the Basic Stereo Consistency Event (BSCE) allows to specify semantically which neighbours have positive and negative contributions to the confidence. It was shown that it could improve significantly the reliability of stereo matching.

Moreover, we showed that the same grouping relation can be used to interpolate contours between pairs of linked primitives. This was then used to correct primitives with the contour as interpolated from its neighbours. In 2D, we obtained a reduction by more than 30% of the orientation error, and more than 10% for the phase. When interpolating 3D primitives, we additionally found that the localisation error was reduced by more than 20%, and the orientation error by more than 15%. Therefore, this interpolation step proved to be a robust manner to improve the representation accuracy, both in 2D and 3D. Because the scheme is local, there is no *a priori* assumption that the whole contours comply with a certain mathematical description: we only assume that the contour is smooth between two proximate primitives, and model this using Hermite interpolation.

Finally, we showed that using such mutual feedback between mid–level, local processes allow to disambiguate them without need for additional contextual knowledge. Thereby, we provide a reliable 3D representation of the shapes in the scene that can then be used for higher level visual operations, where contextual knowledge may be available. This framework was used successfully to address a variety of robot vision tasks: e.g., grasping [13], ego–motion estimation [61], and learning of objects' shapes [12].
